# Recent Progress in Characterizing Long Noncoding RNAs in Cancer Drug Resistance

**DOI:** 10.7150/jca.30877

**Published:** 2019-10-22

**Authors:** Wenyuan Zhao, Bin Shan, Dan He, Yuanda Cheng, Bin Li, Chunfang Zhang, Chaojun Duan

**Affiliations:** 1 Department of Oncology, Xiangya Hospital, Central South University, Changsha, PR China;; 2 College of Medical Sciences, Washington State University Spokane, WA, USA;; 3 Hunan Cancer Hospital, The Affiliated Tumor Hospital of Xiangya Medical College, Central South University, Changsha, PR China;; 4 Department of Thoracic Surgery, Xiangya Hospital, Central South University, Changsha, PR China;; 5 Institute of Medical Sciences, Xiangya Hospital, Central South University, Changsha, PR China;; 6 National Clinical Research Center for Geriatric Disorders, Xiangya Hospital, Central South University, Changsha, PR China.

**Keywords:** lncRNA, cancer, drug resistance, chemotherapy, exosome

## Abstract

Drug resistance is an important cause of failure in cancer chemotherapies. A large number of long noncoding RNAs (lncRNAs) have been found to be related to drug resistance in cancers. Therefore, lncRNAs provide potential targets for cancer therapies. The lncRNAs involved in cancer drug resistance are attracting interest from an increasing number of researchers. This review summarizes the latest research on the mechanisms and functions of lncRNAs in cancer drug resistance and envisages their future developments and therapeutic applications. This research suggests that lncRNAs regulate drug resistance through multiple mechanisms. LncRNAs do not affect drug resistance directly; usually, they do so by regulating the expression of some intermediate regulatory factors. In addition, lncRNAs exhibit a diversity of functions in cancer drug resistance. The overexpression of most lncRNAs promotes drug resistance, while a few lncRNAs have inhibitory effects.

## Introduction

Currently, cancer has become a major threat to human health with increasing mortality. Many cancer patients are in an advanced stage, and few of them are cured by operations. Chemotherapy, as the main treatment for middle-late cancers, can significantly enhance survival rates. However, drug resistance is a major obstacle to the success of cancer chemotherapies. Many studies have focused on finding effective therapeutic targets for cancer drug resistance. LncRNAs, which were previously regarded as transcriptional noise, have been found to be associated with gene expression regulation concerning drug resistance 1. In addition, lncRNAs are also involved in cell proliferation, differentiation and tumor development.

It is widely accepted that only 2% of the human genome participates in protein coding, while the remaining 98% is transcribed into noncoding RNAs (ncRNAs) 2, 3, which can be classified as small ncRNAs and lncRNAs based on their size. Small ncRNAs are those smaller than 200 nucleotides, such as microRNA (miRNA) and small interfering RNA (siRNA). LncRNAs are larger than 200 nucleotides and lack the important open reading frames 4. LncRNAs are mostly distributed in the nucleus and are highly similar to protein-coding RNAs in the aspects of synthesis and processing. The similarity is attributable to the fact that Polymerase II also transcribes lncRNAs, which generally have fewer exons and lower expression levels than mRNAs. In addition, lncRNAs are more cell type specific and less conserved than mRNAs. Most lncRNAs contain a proximal promoter sequence, intronic or exonic sequences, and a secondary RNA structure 5.

The diversity of lncRNAs suggests potential multifunctions associated with normal physiological processes in cells. It has been reported that lncRNAs play a significant role in many life activities, such as dosage compensation effect, cell cycle regulation, epigenetic regulation and cell differentiation regulation 6. Moreover, lncRNAs at different locations have different functions. For example, nuclear lncRNAs take part in transcriptional regulation, RNA processing and chromatin interaction 7. Cytoplasmic lncRNAs are involved in the stability and translation of mRNAs and affect cell signaling 8.

Exploring the drug-resistant mechanisms and functions of lncRNAs in cancers has become a heavily researched subject in the bioscience field. It has been confirmed that ectopic expression of lncRNAs can result in malignant transformation and some common cancers, such as breast cancer, liver cancer and lung cancer, by interacting with DNA, RNA, and protein. However, although thousands of lncRNAs have been identified, their relationship with cancer drug resistance requires further research. In this review, we summarize the recent progress in research examining the role of lncRNAs in the drug resistance of cancer, including their mechanisms and functions, and discuss the future prospects of using lncRNAs as potential targets for cancer therapy.

### Mechanisms of cancer drug resistance related to lncRNAs

Drug resistance can be roughly divided into intrinsic resistance and acquired resistance based on occurrence mechanisms 9, 10. Intrinsic resistance means that resistance factors have already existed in cancer cells before chemotherapy, while acquired resistance results from gene mutations or the acquisition of exogenous resistance genes during treatment. Generally, drug resistance mechanisms that relate to tumor proliferation and prevention of tumor cell death include increased rates of drug efflux, apoptosis inhibition, alterations in the tumor microenvironment and metabolism, emergence of cancer stem cells, and mutations of drug targets 11. In this section, the mechanisms of cancer drug resistance related to lncRNAs are summarized. Table 1 lists the lncRNAs that have recently been determined to be associated with cancer drug resistance and outlines their regulating targets/pathways.

## 1. Promoting cell proliferation

Since cell proliferation is closely associated with drug resistance, lncRNA LUCAT1 promotes methotrexate resistance in osteosarcoma by regulating the miR-200c/ABCB1 axis. In addition, LUCAT1 enhances the expression of the genes associated with drug resistance and promotes the proliferation and invasion of osteosarcoma cells 12. In contrast to LUCAT1, lncRNA FENDRR sensitizes osteosarcoma cells to doxorubicin by suppressing ABCB1 and ABCC1 13. Bian et al. found that UCA1 improves cell proliferation and drug resistance to 5-FU in CRC by suppressing miR-204-5p 45. In addition, there is a UCA1-miR-204-5p-CREB1/BCL2/RAB22A regulatory network in CRC. Furthermore, lncRNA CRNDE promotes the proliferation and drug resistance of CRC cells by miR-181a-5p mediating Wnt/β-catenin signaling. CRNDE also mediates LIMK2b by regulating the enhancer of zeste homolog 2 (EZH2), which promotes drug resistance in CRC 14.

### 1.1. Regulating drug efflux

Drug efflux alteration is the most common mechanism for cancer drug resistance. With increasing drug efflux, the intracellular drug concentration grows lower than the cell death threshold, thereby causing drug resistance. The expression levels of transporters, including ATP-binding cassette (ABC) protein, multidrug resistance associated protein (MRP) and copper transport protein (CTP), are related to drug efflux and are affected by various lncRNAs 15.

Downregulating the expression of lncRNA H19 lowers the cellular drug accumulation level in human hepatocellular carcinoma (HCC) by enhancing the methylation of the multidrug resistant gene 1 (MDR1) promoter and upregulating p-glycoprotein expression, which ultimately leads to doxorubicin resistance. H19 also affects drug resistance in breast cancer via the H19-CUL4A-ABCB1/MDR1 pathway. Experimental results showed that knocking out H19 significantly increased MDR1 and MRP4 in Dox-resistant breast cancer cells, which identified the role of H19 in breast cancer resistance 16.

LncRNAPVT1 was found to be highly expressed in tissues of cisplatin-resistant patients with gastric cancer. In addition, the results of qRT-PCR analysis and western blotting show that PVT1 upregulates the expression of MDR1, MRP, mTOR and HIF-1α, which promotes the development of MDR and suggests a potential target in gastric cancer therapy 17.

### 1.2. Mutations of drug targets

Drug-induced drug resistance is one of the major obstacles that may lead to treatment failure during tumor treatment. Various genetic changes occur when tumor cells divide. In a new generation of tumor cells, some cells may exhibit intrinsic resistance to specific chemotherapeutic drugs. In addition, some tumor cells may carry a gene that produces therapeutic drug-induced resistance 18.

LncRNA BC087858 induces NSCLC resistance to EGFR-TKIS by a non-T790M mutation and activates the PI3K/AKT and MEK/ERK pathways and EMT 19. LncRNA BC087858 knockout restored the sensitivity to gefitinib and suppressed the activation of the PI3K/AKT and MEK/ERK pathways and EMT by promoting the expression of ZEB1 and snails. At the same time, UCA1 expression was significantly upregulated in lung cancer patients with gefitinib resistance 20. Furthermore, overexpression of UCA1 was associated with shorter survival in lung cancer. By activating the AKT/mTOR pathway and EMT, mTOR effectively changes the expression of UCA1, which restored gefitinib sensitivity in acquired resistance cells with non-T790M mutations.

Mutation is a non-negligible resistance mechanism. However, the relationship between lncRNA and mutations in drug resistance remains to be discovered.

### 1.3. Arresting cell cycle

DNA damage repair and warding off cell death induce cell cycle arrest, which allows repairing the damage. The mammalian cell cycle is precisely controlled by cyclin-dependent kinases (CDKs) and related pathways, such as the pRB and p53 pathways. In cancer, cell cycle arrest is disrupted by gain- and loss-of-function mutations, such as in p53 21. Recent studies have shown that many lncRNAs are involved in the regulation of key regulators of the cell cycle, such as cyclins, CDKs, CDK inhibitors, pRB and p53 22. These lncRNAs play a role in epigenetic regulation, transcription factor regulation, posttranscriptional regulation and protein scaffolding. These lncRNAs control the levels of cell cycle regulators through a variety of mechanisms, possibly providing diversity and reliability for the general cell cycle.

Current research indicates that CDKs can regulate cell cycle, cell proliferation and apoptosis, and other biological processes, but few studies have investigated CDK-related cell cycle arrest and drug resistance. CDKs and their associated pathways (pRB and p53) may represent a potential research direction in the field of drug resistance.

Interestingly, blocking CDK can induce cell cycle arrest, which reduces the curative effect of chemotherapy. It has been reported that inhibiting lncRNA NONHSAT028712 significantly reduces CDK2 mRNA levels and induces cell cycle arrest in G1 phase. In addition, receptor tyrosine kinase (RTK)/RAS signaling and cyclin E1 play important roles in CDK2 inhibitor resistance by activating E2F and ETS 23. It can be inferred that the ETS factor ETV5 may be the downstream target of E2F.

### 1.4. Inducing EMT

Epithelial-mesenchymal transition (EMT) is a process in which epithelial cells are connected to the basement membrane, lose their polarity and intercellular adhesion ability, and ultimately become ectomesenchymal cells. This change in biological characteristics enables cells to migrate and invade. It is widely accepted that EMT is essential for stemness, tumor progression, metastasis and drug resistance. Chemotherapy has been shown to be a main inducer of EMT, eventually leading to secondary resistance in cancer cells. It has also been reported that lncRNA MALAT1 increases oxaliplatin resistance in CRC by promoting EMT 24, 25. Suppressing the expression of lncRNA SLC25A25-AS1 promotes EMT and drug resistance in CRC 26. This effect indicates that the curative effect of chemotherapy can also be enhanced by upregulating certain lncRNAs.

### 1.5. Promoting glycometabolism

**Figure 1:**
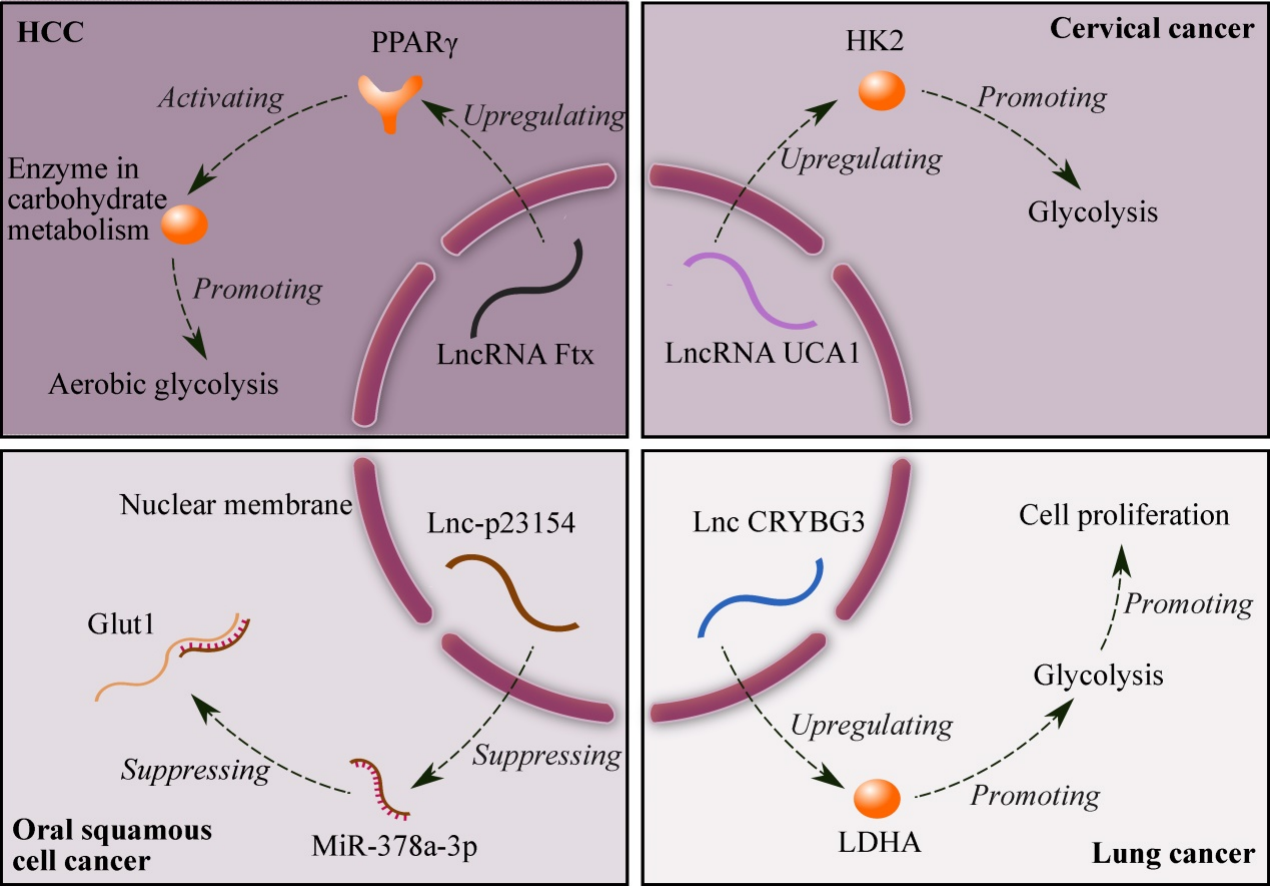
Mechanisms of glycolysis regulated by four lncRNAs. Symbols: brown ellipses - enzymes; brown Y shape - receptor (PPARγ).

Suppressing glycolysis significantly promotes apoptosis in drug-resistant cancer cells 27. Fig. 1 shows four lncRNAs related to glycolysis: (1) lncRNA Ftx promotes the expression of peroxisome proliferator-activated receptor γ (PPARγ), and PPARγ upregulates the expression of enzymes in carbohydrate metabolism, which ultimately enhances aerobic glycolysis in HCC. In addition, the alterations in lactate production, glucose uptake and the enzyme expression induced by Ftx can be counteracted by downregulating the expression of PPARγ 28; (2) lncRNA UCA1 promotes glycolysis by upregulating hexokinase 2 (HK2) in cervical cancer 29; (3) lncRNA-p23154 suppresses the expression of miR-378a-3p, and miR-378a-3p can promote Glut1-mediated glycolysis in oral squamous cell cancer by binding and inhibiting Glut1 30; and (4) lncRNA CRYBG3 increases the expression of lactate dehydrogenase A (LDHA), and LDHA promotes glycolysis in lung cancer. Furthermore, glycolysis can enhance cell proliferation 31. Therefore, lncRNAs can be potential targets for cancer therapy due to their effects on glycolysis.

## 2. Cell death

### 2.1. Dual regulation of autophagy

Autophagy is the main cause of chemotherapeutic failure in non-small-cell lung cancer (NSCLC) and can be regulated by lncRNAs. For example, lncRNA XIST significantly decreases autophagy by regulating ATG7, which indicates that XIST may be a potential target for cisplatin chemotherapy of NSCLC. In addition, the XIST/miR-17/autophagy pathway may also be a promising target 32. Besides, lncRNA AC023115.3 suppresses drug resistance in glioblastoma by inhibiting autophagy 33.

Autophagy has dual characteristics of tumor suppressor and carcinogen and is regulated by various signaling pathways, such as PI3K-Akt-m TOR, Beclin1, BCL-2, Ras and p53. A recent study showed that the overexpression of lncRNA HOTAIR increases drug resistance in NSCLC by promoting autophagy through inhibiting the phosphorylation of ULK1. Similarly, upregulating lncRNA CASC2 can sensitize glioma to the cytotoxicity of temozolomide by suppressing autophagy 34. However, lncRNA XIST increases chemotherapy resistance in NSCLC cells by inhibiting autophagy 32. In addition, lncRNA HULC induces autophagy in HCC by stabilizing silent information regulator 1 (Sirt1). As a result, this lncRNA enhances drug resistance to three anti-cancer drugs: oxaliplatin, pirarubicin and 5-FU. It is also revealed that the drug resistance in HCC can be increased through the HULC/USP22/Sirt1/protective autophagy pathway 35.

### 2.2. Suppressing apoptosis

Since most drugs prevent cancer cell growth by promoting apoptosis, suppressing apoptosis may induce drug resistance and accelerate the proliferation of cancer cells. It has been reported that lncRNAs can increase apoptosis-induced drug resistance by upregulating survival factors, such as BCL-2, nuclear factor kappa B, and inhibitor of apoptosis protein 36. Additionally, lncRNA HOTTIP enhances drug resistance in small cell lung cancer (SCLC) by promoting BCL-2 expression 37. Moreover, UCA1 increases cisplatin resistance by upregulating Wnt6 expression and suppressing cell apoptosis in bladder cancer 38.

The overexpression of HOTAIR improves gastric cancer cell proliferation and cell cycle G1/S transition. In addition, HOTAIR overexpression decreases cancer cell apoptosis, which can activate the P13K/AKT/MRP1 genes by targeting miR-126. As a result, cisplatin resistance in gastric cancer is increased 39. LncRNA GAS5 is closely associated with doxorubicin resistance in bladder transitional cell cancer (BTCC) and inhibiting proliferation. The expression of this lncRNA is suppressed in UBC and increases with the pathological grades of BTCC. The overexpression of GAS5 promotes apoptosis by decreasing the expression of the anti-apoptosis protein BCL-2. Similarly, UCA1 promotes cisplatin resistance in bladder cancer by increasing the expression of Wnt6 40.

Besides, lncRNA MEG3 regulates cisplatin (DDP) resistance in NSCLC by acting as a competing endogenous RNA. MEG3 can interact with miR-21-5p directly and decrease its expression, and miR-21-5p can significantly eliminate the effects of MEG3 on DDP resistance by regulating cell proliferation and apoptosis. Moreover, SOX7 is a direct target for MEG3 and miR-21-5p and upregulating the miR-21-5p/SOX7 axis can reverse the pro-proliferative and anti-apoptotic effects induced by knocking out MEG3. This finding helps to elucidate the molecular mechanism of DDP resistance associated with MEG3 in NSCLC 41.

MEG3 promotes chemosensitivity by enhancing apoptosis induced by oxaliplatin. However, Li et al. found that lncRNA TUG1 works as a competing endogenous RNA (ceRNA) to sponge miR-186, and miR-186 targets CPEB2 directly in CRC 42.

The overexpression of H19 can increase methotrexate resistance in colorectal cancer by inducing EMT. In addition, lncRNA PVT1 promotes EMT by upregulating the expression of the transcription factor Twist1 through the sponge effect 43.

### 2.3. Microenvironmental changes

**Figure 2:**
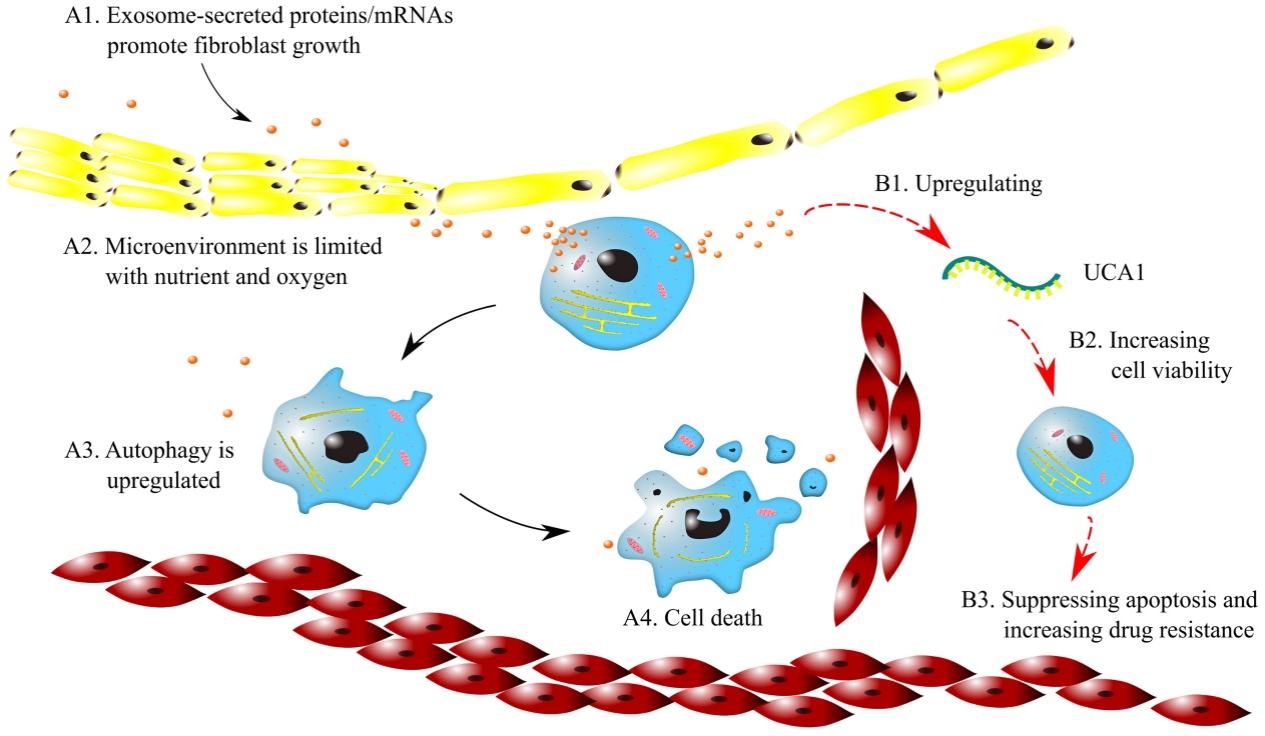
Mechanisms of drug resistance regulated by exosomes in microenvironments. Symbols: red cells - red blood cells; blue cells - cancer cells; yellow cells -fibroblast; brown dots - exosomes; green curve - lncRNA UCA1; black arrows - for A1~A4; red arrows - for B1~B3.

Microenvironmental changes can lead to drug resistance in cancers. It was found that the large oxygen consumption of cancer cells causes hypoxic microenvironments in solid tumors. Therefore, cancer cells switch to anaerobic respiration, which results in persistent acidic microenvironments. Hypoxic and acidic microenvironments can lead to genetic instability and activate signaling pathways 44. Emerging evidence shows that exosomes affect tumor-associated pathways in microenvironments, including angiogenesis, cancer stemness, cell metastasis and EMT driven by hypoxia 45. For example, as shown in Fig. 2 (A1~A4), exosomes secrete proteins and mRNAs, which induce a desmoplastic reaction (DR) and promote fibroblast growth. As a result, the circulation of anticancer drugs is blocked, and drug resistance is acquired. Furthermore, since tumor nodes are often not close to capillaries, tumor microenvironments are limited in oxygen and essential nutrients. This limitation upregulates autophagy and suppresses cell proliferation, which may contribute to the resistance to cycle-active anticancer drugs.

Exosomes promote drug resistance to tamoxifen in breast cancer by regulating the transfer of UCA1. The exosomes from tamoxifen-resistant cells contain notably more UCA1s than those from tamoxifen-sensitive cells. Knocking out UCA1 can decrease tamoxifen resistance mediated by exosomes. In addition, H19 regulates drug resistance in ERα-positive breast cancer by epigenetically silencing the pro-apoptotic gene BIK 46. As illustrated in Fig. 2 (B1~B3), the exosomes released from breast cancer cells increase the expression of UCA1, which enhances cell viability. Consequently, apoptosis is suppressed, and drug resistance can be increased.

## 3. Other mechanisms

### 3.1. Cancer stemness

The expression of lncRNA LET is suppressed in urinary bladder cancer (UBC) after gemcitabine treatment. The overexpression of TGFβ1 and a low level of lncRNA LET and miR-145 can predict poor prognosis. TGFβ1 also promotes gemcitabine resistance by downregulating the signaling of the LET/NF90/miR-145 axis. In addition, TGFβ1 can increase drug resistance in UBC by promoting cancer stemness 47.

### 3.2. Epigenetic modification

Recent studies have shown that lncRNAs are greatly involved in drug resistance by epigenetic modification, especially methylation. For example, lncRNA HOTAIR suppresses HOXA1 methylation by inhibiting DNMT1 and DNMT3B, which leads to chemoresistance in SCLC 48. H19 promotes p-glycoprotein overexpression and drug resistance in HCC by regulating the promoter methylation of MDR1 49. It is worthwhile to investigate the relationship between drug resistance and gene methylation affected by lncRNAs. LncRNA can affect cell proliferation by mediating methylation in pancreatic cancer. For example, lncRNA HOTAIR regulates EZH2 and miR-34a by mediating the methylation of H3K27 and suppresses the miR-663b promoter by altering histone methylation, and EZH2, miR-34a and miR-663b can affect cell proliferation 50.

Phosphorylation is an important factor for drug resistance based on genetic modification. The overexpression of HOTAIR reduces the sensitivity to cisplatin by activating STAT3 and increasing the expression of ABCB1. In addition, promoting the phosphorylation of STAT3 suppresses ABCB1 directly, which results in chemoresistance to cisplatin in HCC 51. Further research is warranted to determine the mechanisms of cancer drug resistance associated with phosphorylation.

### 3.3. Chinese herbal medicines

**Figure 3:**
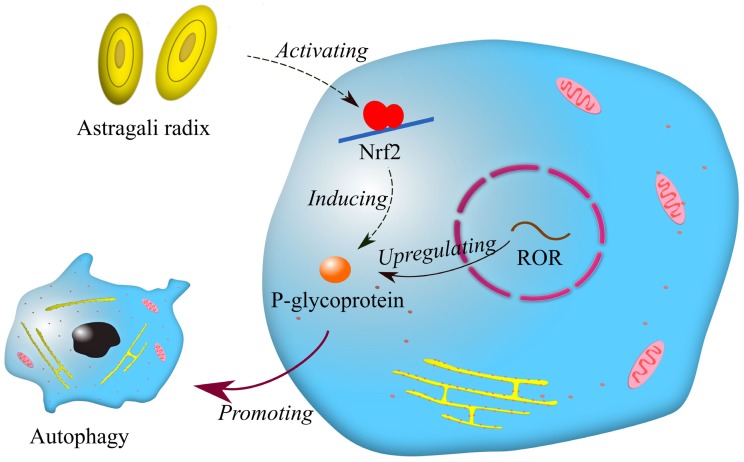
Mechanisms of drug resistance regulated by astragali radix and lncRNA ROR. Symbols: blue cells—breast cancer cells; red circle—nuclear membranes; brown dots—enzymes; yellow net—endoplasmic reticulum; pink ellipses—mitochondria.

In the last ten years, extracts from Chinese herbal plants, as a natural addition, have been commonly employed in the development of new anti-cancer drugs because of their mildness and effectiveness. It has been reported that p-glycoprotein is associated with multidrug resistance in breast cancer 52. Afterwards, Li et al. found that lncRNA ROR increases the expression of p-glycoprotein, and p-glycoprotein is involved in drug resistance in breast cancer by promoting autophagy 53. Interestingly, a recent report showed that astragali radix promotes the expression of p-glycoprotein by activating Nrf2 54. It can be inferred that astragali radix may be related to lncRNA ROR in drug resistance of breast cancer (Fig. 3). Astragali radix is a famous Chinese herbal medicine widely used in traditional Chinese medicine prescriptions. Although it was previously believed that traditional Chinese medicine had little effect on treating cancers, it is now reasonable to think that traditional Chinese medicine may benefit cancer therapy by regulating drug resistance.

## 4. Conclusions

This review summarizes the drug-resistance mechanisms of lncRNAs in cancers. The review contains not only the typical mechanisms, that is, drug efflux, mutations of drug targets, arresting cell cycle, EMT, autophagy and apoptosis, but also newly discovered mechanisms, including glycolmetabolism, microenvironmental changes, cancer stemness, genetic modification and Chinese herbal medicines. Obtained data indicate that lncRNAs are involved in cancer drug resistance by regulating some intermediate regulatory factors. In addition, this review lists many lncRNAs that have recently been found to be related to cancers and their regulatory pathways. The functions of lncRNAs in cancer drug resistance are described in detail according to cancer types. A certain lncRNA can regulate the drug resistance of multiple cancers, and a certain kind of cancer can be associated with several lncRNAs. Finally, the overexpression of most lncRNAs enhances drug resistance in cancers, while a few of them can sensitize the cells to anticancer drugs. This effect demonstrates the functional diversity of lncRNAs, which requires further research. In addition, it is necessary to further elucidate the functions of more lncRNAs in cancer drug resistance and their mechanisms, which will provide more targets for cancer therapies.

### Future prospects

Although many studies have focused on the functions and mechanisms of lncRNAs in cancer drug resistance, a large proportion of them remain unrevealed and warrant further investigation. Future drugs for cancer chemotherapies can be combinations of existing anticancer drugs and drugs targeting lncRNAs related to drug resistance. Besides, we anticipate that some studies can be conducted to identify the basic drug-resistant mechanisms associated with lncRNAs, which may provide some common targets for cancer therapies. Moreover, the finding that astragali radix is involved in cancer drug resistance may lead to more research on the relations of traditional Chinese medicines and the drug resistance related to lncRNAs.

## Figures and Tables

**Table 1:** LncRNAs that have been or might be linked to cancer drug resistance

Cancer type	LncRNAs	Drug resistance	Function
**Breast cancer**	H19 46	Paclitaxel	Silencing of the pro-apoptotic gene BIK
	UCA1 55	Doxorubicin	Suppressing the p27 protein level and promoting cell growth and tumorigenesis
	HOTAIR 56	Tamoxifen	Interacting with ER and improving transcriptional activities of ER; its overexpression upregulates breast carcinoma cell proliferation
	CCAT2 57	Tamoxifen	Promoting cell migration
	BCAR4 58	Tamoxifen	NA
	ROR 59	Tamoxifen	Induces autophagy
	ATB 60	Trastuzumab	Promotes cell proliferation, EMT, invasion and metastasis
**Gastric cancer**	MRUL 61	Vincristine	Enhancing ABCB1 expression
	BCAR4 62	Cisplatin	NA
	HNRNPC 63	5-fluorouracil Paclitaxel Cisplatin	Its high expression level indicates poor overall survival and free of progression
	AK002798 64 PVT1 17	Paclitaxel Cisplatin Cisplatin	Increasing cell apoptosis and the expression of P-glycoprotein and MRP1; decreasing the expressions of Caspase 3 and Caspase 8 Overexpression of LncRNA PVT1 in gastric carcinoma promotes the development of MDR
**Bladder cancer**	CUDR 65	Cisplatin	Downregulating VEGF/PI3K/Akt signaling pathway
	UCA1 66	Cisplatin/gemcitabine	Modulating miR-196a-5p of bladder carcinoma cells
	NCRAN 67	Cisplatin	Upregulating drug resistance; suppressing apoptosis
	LET/NF90/miR-145 68	Gemcitabine	Upregulating TGFβ1
	GAS5 69	Doxorubicin	Promoting apoptosis; depressing the expression of anti-apoptosis protein Bcl-2
**Lung cancer**	HOTAIR	Crizotinib 70	Activating autophagy by inhibiting the phosphorylation of ULK1
		Gefitinib 71	Activating Bax/Caspase-3 and TGF-α/EGFR signaling
	AK126698 72	Cisplatin	Suppressing canonical Wnt signaling pathway; downregulating cisplatin resistance
	MEG3 41	Cisplatin	Enhancing cisplatin sensitivity via regulating miR-21-5p/SOX7 axis; activating p53 and Bcl-xl of LAD cells
	XIST 32	Cisplatin	Enhancing the drug resistance of NSCLC cells via suppressing autophagy
	AK001796 73	Cisplatin	Upregulating the expression of ANRIL
	KCNQ1OT1 74	Paclitaxel	Upregulating the chemoresistance
	MIAT 9	Gefitinib	Regulating miR-34a
	TUG1 75	Cisplatin	Regulating LIMK2b via EZH2
	ANRIL 76	Cisplatin	Inhibits proliferation, induces apoptosis
**Colorectal cancer**	H19 77	Doxorubicin	Overexpression of H19 activating Wnt/β-catenin pathway
	CUDR 78	5-fluorouracil	Upregulating miR-195
		Oxaliplatin	Regulating Wnt/β-catenin signaling via MiR-181a-5p
	SNAR 79	5-Fluorouracil	Suppressing the drug resistance to 5-FU
	Lnc00152 80	Oxaliplatin	Functioning as a competing endogenous RNA
	SLC25A25-AS1 81 PCTA-1 43	Cisplatin 5-FU	Downregulating SLC25A25-AS1 to promote proliferation Upregulating the chemoresistance
**Hepatocellular carcinoma**	H19 15	Doxorubicin	Inducing the expression of P-glycoprotein and the drug resistance of MDR1; regulating MDR1 promoter methylation
	Lnc-VLDLR 82	Sorafenib, Camptothecin, Doxorubicin	Promoting cell-cycle progression; knocking out it suppresses ABCG2 expression
	HULC 35	Oxaliplatin, 5-Fluorouracil Pirarubicin	Stabilizing Sirt1
	ARSR 83	Doxorubicin	Promoting PTEN mRNA degradation; suppressing PTEN expression; activating p13k/AKt pathway
**Osteosarcoma**	LUCAT1 84	Methotrexate	Regulating drug resistance through miR-200c/ABCB1 axis
	FENDRR 85	Doxorubicin	Downregulating ABCB1 and ABCC1
	ODRUL 86	Doxorubicin	Downregulation of ODRUL partly suppresses the drug resistance to doxorubicin
	NR_036444 87	Doxorubicin	NA
	FOXC2-AS1 88	Doxorubicin	Increases the expression of FOXC2
	Lnc00161 89	Cisplatin	Induces apoptosis regulating the miR-645-IFIT2 axis
**Glioblastoma**	AC023115.3 90	Cisplatin	Reducing autophagy
	MALAT1 25	Temozolomide	Promoting microRNA-101
**Human squamous carcinoma**	CUDR 91	5-Fluorouracil	Causing a drug-induced apoptosis of cells by EMT and cancer stem cell-like properties
**Cervical cancer**	GAS5 92	Cisplatin	Suppressing tumor via microRNA 21,
**Cancer-associated fibroblasts**	ANRIL 93	Cisplatin	Upregulating the expression of ANRIL
**Squamous cell carcinoma**	EGFR-AS1 94	Gefitinib	Mediating epidermal growth factor receptor addiction and modulating treatment response
**Endometrial cancer**	LncC00672 95	Paclitaxel	Contributing to gene suppression mediated by p53 protein and promoting the chemosensitivity of endometrial cancer
**Ovarian cancer**	ENST00000457645 96	Cisplatin	Modifying apoptotic protein expression
	HOTAIR 97	Carboplatin	DNA methylation
**Cholangiocarcinoma**	NEAT-1 98	Gemcitabine	BAP1 overexpressing
**Nasopharyngeal carcinoma**	N375709 99	Paclitaxel	NA
**Esophageal squamous cell carcinoma**	AFAP1-AS1 100	Cisplatin	Upregulating
**Renal cancer**	SRLR 101	Sorafenib	Evokes IL-6/STAT3 axis

## Abbreviations

LncRNAs: long noncoding RNAs
ncRNAs: noncoding RNAs
miRNA: microRNA
siRNA: small interfering RNA
MRP: multidrug resistance associated protein
ABC: ATP-binding cassette
CTP: copper transport protein
HCC: hepatocellular carcinoma
MDR1: multidrug resistant gene 1
CDKs: cyclin-dependent kinases
EMT: epithelial-mesenchymal transition
PPARγ: peroxisome proliferator-activated receptor γ
HK2: hexokinase 2
LDHA: lactate dehydrogenase A
NSCLC: non-small-cell lung cancer
Sirt1: silent information regulator 1
SCLC: small cell lung cancer
BTCC: bladder transitional cell cancer
DDP: cisplatin
ceRNA: competing endogenous RNA
DR: desmoplastic reaction
UBC: urinary bladder cancer
